# Crystal structure of an organic–inorganic hybrid compound based on morpholinium cations and a β-type Anderson polyanion

**DOI:** 10.1107/S2056989015019246

**Published:** 2015-10-17

**Authors:** Tamara J. Lukianova, Vasyl Kinzhybalo, Adam Pietraszko

**Affiliations:** a Institute of Low Temperature and Structure Research, Polish Academy of Sciences, Okolna str. 2, PO Box 1410, 50-950 Wroclaw, Poland

**Keywords:** crystal structure, polyoxidomolybdate, Anderson-type anion, organic–inorganic hybrid

## Abstract

A new organic–inorganic hybrid compound, penta­morpholinium hexa­hydrogen hexa­molybdoferrate(III) sulfate 3.5-hydrate, (C_4_H_10_NO)_5_[Fe^III^(OH)_6_Mo_6_O_18_](SO_4_)·3.5H_2_O, was obtained from an aqueous solution. The polyoxidomolybdate (POM) anion is of the Anderson β-type with a central Fe^III^ ion. Three of five crystallographically independent morpholinium cations are disordered over two sets of sites. An intricate network of inter­molecular N—H⋯O and O—H⋯O inter­actions between cations, POMs, sulfate anions and non-coordinating water mol­ecules creates a three-dimensional network structure.

## Abstract 2

The crystal structure of the novel organic–inorganic hybrid compound is based on a β-type Anderson polyoxidomolybdate anion containing a central Fe^III^ ion. In the crystal, inter­molecular N—H⋯O and O—H⋯O hydrogen bonds link the components into a three-dimensional network structure.

## Chemical context   

Polyoxidometalates (POM) are attractive mol­ecular building blocks used in the formation of multidimensional organic–inorganic hybrid networks during self-organization processes (Pope & Müller, 2001 ▸; Müller *et al.*, 1998 ▸; Long *et al.*, 2007 ▸). POMs play an important role in the design of new classes of functionalized materials not only because of their topological versatility and high dimensional architectures, but also due to their rich diversity of remarkable properties. Several related compounds with Anderson-type polyoxidometalate anions and organic cations, such as (C_4_H_12_N_2_)_5_[Al(OH)_6_Mo_6_O_18_]_2_(SO_4_)_2_·16H_2_O (Yang *et al.*, 2009 ▸), (C_4_H_10_NO)_3_[Cr(OH)_6_Mo_6_O_18_]·4H_2_O (Yang *et al.*, 2011 ▸), (C_6_H_10_N_3_O_2_)_2_Na(H_2_O)_2_[Al(OH)_6_Mo_6_O_18_]·6H_2_O (Thabet *et al.*, 2012 ▸) and other compounds with an Fe^III^ central ion (Marcoux *et al.*, 2003 ▸; Allain *et al.*, 2008 ▸; Dessapt *et al.*, 2011 ▸) have been reported. In β-type Anderson polyoxidoanions, which are characterized by a planar arrangement of the metal atoms, each Mo^VI^ atom has two terminal oxygen atoms, two bridging O atoms and two bridging OH functions which make it highly reactive and easily coordinated by varieties of transition metal atoms in the anion.
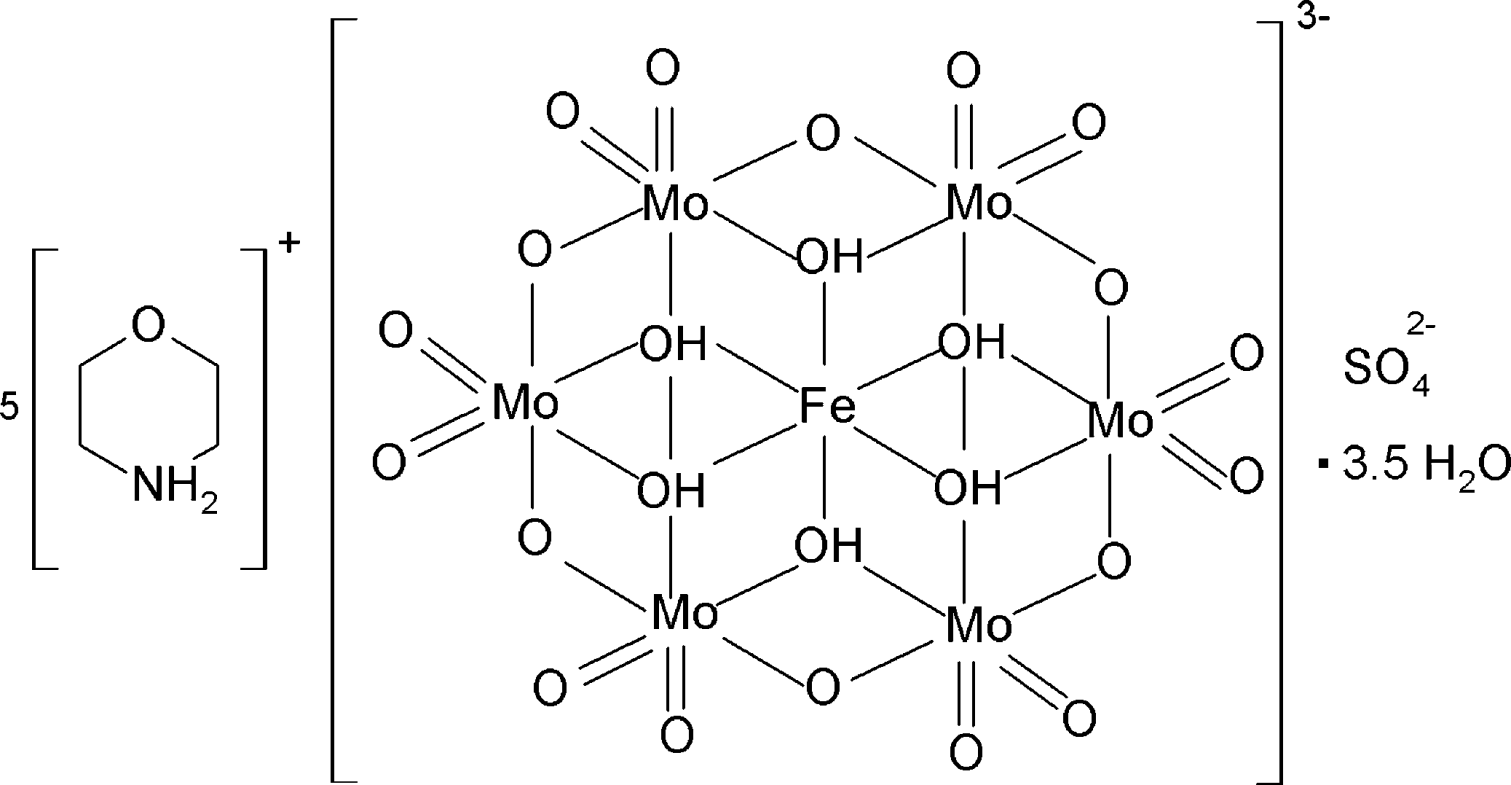


Here we report synthesis and structure of the new organic–inorganic hybrid compound (C_4_H_10_NO)_5_[Fe^III^(OH)_6_Mo_6_O_18_](SO_4_)·3.5H_2_O, (I).

## Structural commentary   

**Figure 1:**
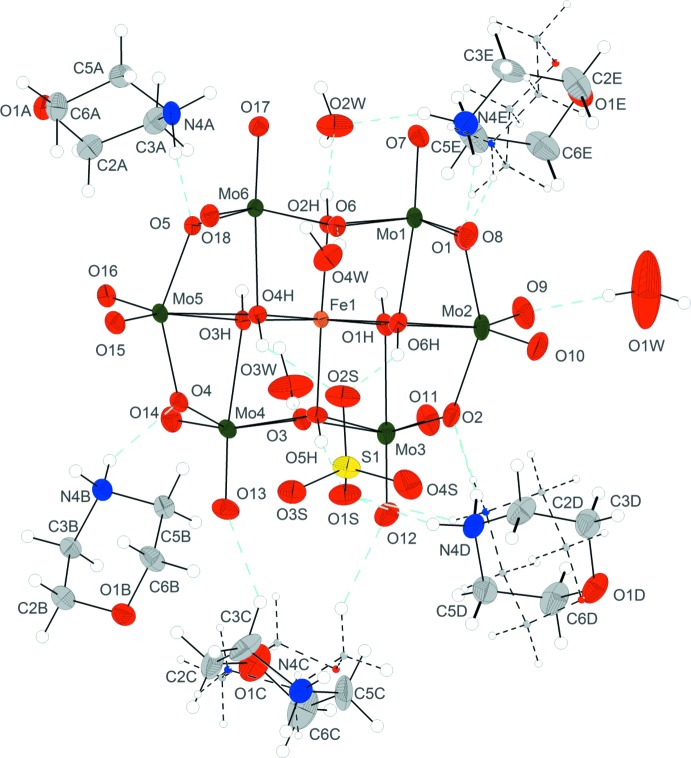
The mol­ecular components in the structure of compound (I). Displacement ellipsoids are drawn at the 45% probability level. Hydrogen bonds are denoted by cyan dashed lines. Minor parts of the disordered cations are shown with dashed bonds.

The asymmetric unit of compound (I) is made up of one Anderson β-type polyoxidoanion, [Fe(OH)_6_Mo_6_O_18_]^3−^, abbreviated in the following as {FeMo_6_}, five morpholinium cations (C_4_H_10_NO)^+^, one sulfate anion and four non-coordinating water mol­ecules (Fig. 1 ▸). Three of the morpholinium cations are disordered over two sets of sites and one water mol­ecule (O1*W*) shows half-occupancy. The {FeMo_6_} anion is formed by six edge-sharing [MoO_6_] octa­hedra, which are arranged hexa­gonally around the central [Fe(OH)_6_] octa­hedron with bond lengths and angles that are within the expected ranges for this type of POM anion (Cao *et al.*, 2007 ▸). The six hydroxyl groups of the Anderson-type polyoxoanion are involved as donor groups in hydrogen-bond formation with O atoms of the sulfate anions and the non-coordinating water mol­ecules.

## Supra­molecular features   

**Figure 2:**
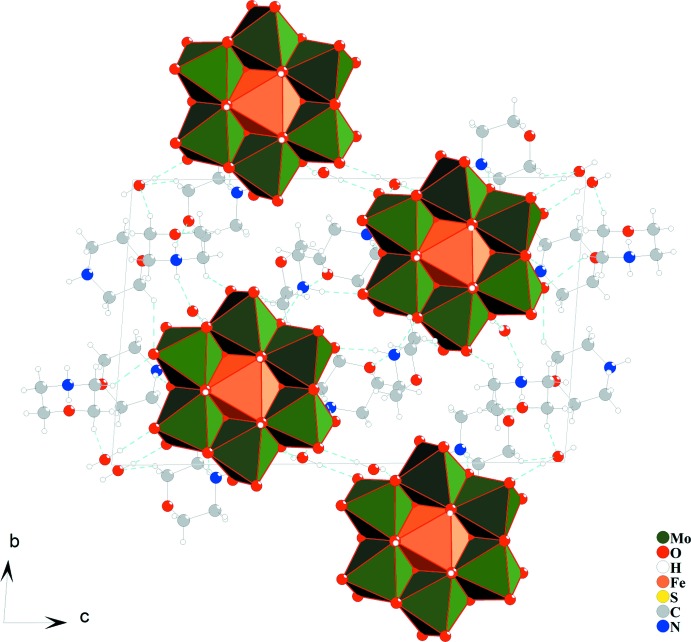
The crystal packing of compound (I) in a projection along [100], shown in the polyhedral mode for the POM anion. Orange and green octa­hedra are [FeO_6_] and [MoO_6_], respectively. Hydrogen bonds are shown as cyan dashed lines. Minor components of disorder for the morpholinium cations are omitted for clarity.

In the crystal structure of compound (I), hydrogen-bonding inter­actions between morpholinium cations, polyoxidoanions, sulfate anions and non-coordinating water mol­ecules are of the types O—H⋯O and N—H⋯O (Table 1 ▸) and connect the discrete units into a three-dimensional supra­molecular network. Hydrogen bonding is the dominating inter­molecular inter­action involved in the construction of this architecture and gives sufficient stabilization of its crystal structure. Figs. 2 ▸ and 3 ▸ shows the crystal packing with hydrogen-bonding inter­actions.

## Synthesis and crystallization   

The title compound was synthesized by the following procedure: 0.320 g (0.8 mmol) of iron(III) sulfate was dissolved in 10 ml of double-distilled water. To this solution 4 ml (5 mmol) of morpholine and 0.309 g (1.5 mmol) of Na_2_MoO_4_ were added during constant stirring. By the addition of 30%_wt_ sulfuric acid, the pH was adjusted to 2.5. The resultant solution was filtered and the filtrate kept at room temperature. After few weeks, light-brown crystals were obtained.

## Refinement   

Crystal data, data collection and structure refinement details are summarized in Table 2 ▸. Three of the five crystallographically independent morpholinium cations are disordered, for which all atoms are distributed between two positions. The refined site occupation factor ratios are as follows: 0.857 (6):0.143 (6), 0.703 (9):0.297 (9) and 0.857 (6):0.143 (6) for O1*C*–C6*C*/O11*C*–C61*C*, O1*D*–C6*D*/O11*D*–C61*D* and O1*E*–C6*E*/O11*E*–C61*E*, respectively. All non-hydrogen atoms were refined anisotropically, except for the minor parts of the disordered morpholinium cations. The positions of the H atoms were initially located in difference Fourier maps. All H atoms were fixed at ideal positions, with *U*_iso_(H) = 1.2*U*_eq_ of the parent atom (1.5*U*_eq_ for water H atoms). In the final refinement cycles, H atoms of the O1*W* water mol­ecule were allowed to ride on the parent O atom (AFIX 3 in *SHELXL2014*; Sheldrick, 2015 ▸), H atoms of the other water mol­ecules were fixed with the AFIX 6 instruction. For the minor component of disorder for morpholinium cation (O11*C* > C61*C*) the SAME instruction was used. Pairs of morpho­lin­ium cations (labelled *C* and *E*) were initially refined with individual occupation factor variables which turned out to refine to the same value (taking into account standard uncertainties). As a result of the fact that disordered parts of these two cations are connected by hydrogen-bonding inter­actions, disorder was restrained with a common occupation factor variable in the final refinement. One of the O atom of a water mol­ecule (O1*W*) is characterized by a significantly elongated displacement parameter. The occupation factor of this mol­ecule was arbitrarily fixed at 50%.

## Supplementary Material

Crystal structure: contains datablock(s) I. DOI: 10.1107/S2056989015019246/wm5221sup1.cif

Structure factors: contains datablock(s) I. DOI: 10.1107/S2056989015019246/wm5221Isup2.hkl

CCDC reference: 1430684

Additional supporting information:  crystallographic information; 3D view; checkCIF report

## Figures and Tables

**Figure 3:**
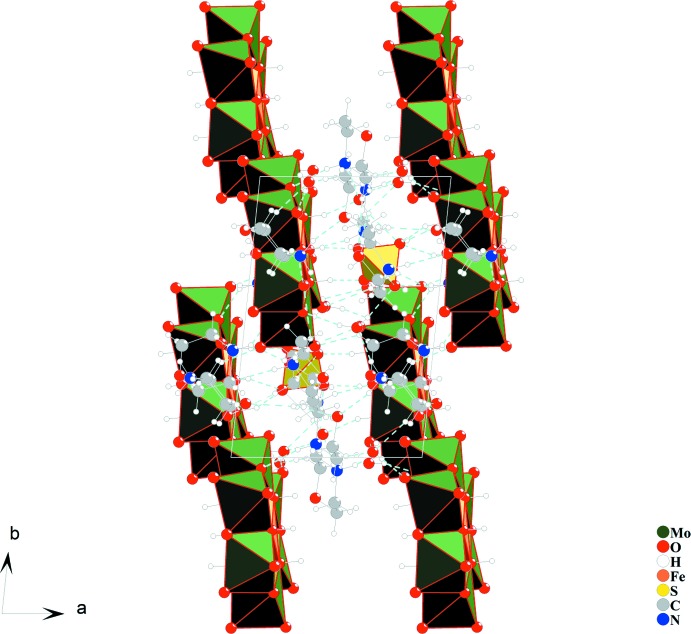
The crystal packing of compound (I) in a projection along [001].

**Table 1:** Hydrogen-bond geometry (, )

*D*H*A*	*D*H	H*A*	*D* *A*	*D*H*A*
O1*W*H1*WA*O9	0.84	1.94	2.781(11)	178
O1*W*H1*WB*O10^i^	0.85	1.99	2.831(11)	173
O2*W*H2*WA*O4*W* ^ii^	0.85	1.80	2.619(4)	161
O2*W*H2*WB*O2*S* ^ii^	0.85	2.05	2.876(4)	164
O2*W*H2*WB*O4*S* ^ii^	0.85	2.48	3.129(5)	133
O3*W*H3*WA*O1*S* ^ii^	0.85	1.99	2.790(5)	155
O3*W*H3*WB*O3	0.85	1.92	2.753(4)	167
O4*W*H4*WA*O17^iii^	0.85	1.88	2.714(4)	165
O4*W*H4*WB*O6	0.85	1.94	2.761(4)	161
O1*H*H1*H*O4*S* ^ii^	1.00	1.69	2.673(4)	165
O2*H*H2*H*O2*W*	1.00	1.78	2.743(4)	162
O3*H*H3*H*O3*S* ^ii^	1.00	1.61	2.602(4)	174
O4*H*H4*H*O2*S*	1.00	2.13	2.911(4)	133
O5*H*H5*H*O1*S*	1.00	1.69	2.672(4)	165
O6*H*H6*H*O2*S*	1.00	1.83	2.691(4)	142
N4*A*H4*AA*O4*W* ^ii^	0.91	2.34	3.102(5)	141
N4*A*H4*AB*O5	0.91	1.86	2.760(4)	169
N4*B*H4*BA*O4	0.91	2.00	2.761(4)	140
N4*B*H4*BA*O1*A* ^iv^	0.91	2.26	2.840(4)	121
N4*B*H4*BB*O15^v^	0.91	1.97	2.869(4)	171
N4*C*H4*CA*O1*E* ^vi^	0.91	1.97	2.817(7)	155
N41*C*H41*A*O11*E* ^vi^	0.91	1.92	2.54(5)	124
N4*E*H4*EA*O1	0.91	1.91	2.780(6)	160
N41*E*H41*D*O1	0.91	1.52	2.35(3)	150
N4*D*H4*DA*O1*S*	0.91	1.99	2.866(8)	162
N4*D*H4*DB*O2	0.91	2.00	2.846(9)	155
N41*D*H41*E*O2	0.91	1.62	2.520(19)	169

Symmetry codes: (i) 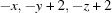; (ii) 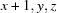; (iii) 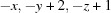; (iv) 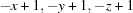; (v) 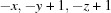; (vi) 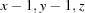.

**Table 2:** Experimental details

Crystal data	
Chemical formula	(C_4_H_10_NO)_5_[Fe(OH)_6_Mo_6_O_18_](SO_4_)3.5H_2_O
*M*_r_	1621.30
Crystal system, space group	Triclinic, *P* 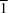
Temperature (K)	100
*a*, *b*, *c* ()	8.900(3), 13.143(4), 20.778(6)
, , ()	84.92(3), 85.37(3), 83.70(3)
*V* (^3^)	2400.1(13)
*Z*	2
Radiation type	Mo *K*
(mm^1^)	1.97
Crystal size (mm)	0.27 0.20 0.12

Data collection	
Diffractometer	Rigaku Oxford Diffraction Xcalibur Atlas
Absorption correction	Analytical (*CrysAlis PRO*; Rigaku Oxford Diffraction, 2015 ▸)
*T*_min_, *T*_max_	0.708, 0.819
No. of measured, independent and observed [*I* > 2(*I*)] reflections	37127, 11708, 9288
*R*_int_	0.038
(sin /)_max_ (^1^)	0.695

Refinement	
*R*[*F* ^2^ > 2(*F* ^2^)], *wR*(*F* ^2^), *S*	0.042, 0.112, 1.02
No. of reflections	11708
No. of parameters	714
No. of restraints	12
H-atom treatment	H-atom parameters constrained
_max_, _min_ (e ^3^)	1.98, 1.29

Computer programs: *CrysAlis PRO* (Rigaku Oxford Diffraction, 2015 ▸), *SHELXS97* (Sheldrick, 2008 ▸), *SHELXL2014* (Sheldrick, 2015 ▸), *DIAMOND* (Brandenburg, 1997 ▸) and *OLEX2* (Dolomanov *et al.*, 2009 ▸).

## supplementary crystallographic information

### Crystal data

**Table:**

(C_4_H_10_NO)_5_[Fe(OH)_6_Mo_6_O_18_](SO_4_)·3.5H_2_O	*Z* = 2
*M**_r_* = 1621.30	*F*(000) = 1608
Triclinic, *P*1	*D*_x_ = 2.243 Mg m^−^^3^
*a* = 8.900 (3) Å	Mo *K*α radiation, λ = 0.71073 Å
*b* = 13.143 (4) Å	Cell parameters from 15587 reflections
*c* = 20.778 (6) Å	θ = 2.4–29.3°
α = 84.92 (3)°	µ = 1.97 mm^−^^1^
β = 85.37 (3)°	*T* = 100 K
γ = 83.70 (3)°	Block, light brown
*V* = 2400.1 (13) Å^3^	0.27 × 0.20 × 0.12 mm

### Data collection

**Table:**

Rigaku Oxford Diffraction Xcalibur Atlas diffractometer	11708 independent reflections
Radiation source: fine-focus sealed X-ray tube, Enhance (Mo) X-ray Source	9288 reflections with *I* > 2σ(*I*)
Graphite monochromator	*R*_int_ = 0.038
Detector resolution: 10.6249 pixels mm^-1^	θ_max_ = 29.6°, θ_min_ = 2.4°
ω scans	*h* = −12→11
Absorption correction: analytical (*CrysAlis PRO*; Rigaku Oxford Diffraction, 2015)	*k* = −18→17
*T*_min_ = 0.708, *T*_max_ = 0.819	*l* = −28→28
37127 measured reflections	

### Refinement

**Table:**

Refinement on *F*^2^	Primary atom site location: structure-invariant direct methods
Least-squares matrix: full	Hydrogen site location: mixed
*R*[*F*^2^ > 2σ(*F*^2^)] = 0.042	H-atom parameters constrained
*wR*(*F*^2^) = 0.112	*w* = 1/[σ^2^(*F*_o_^2^) + (0.0601*P*)^2^ + 2.7906*P*] where *P* = (*F*_o_^2^ + 2*F*_c_^2^)/3
*S* = 1.02	(Δ/σ)_max_ = 0.001
11708 reflections	Δρ_max_ = 1.98 e Å^−^^3^
714 parameters	Δρ_min_ = −1.29 e Å^−^^3^
12 restraints	

### Special details

**Table:**

Geometry. All e.s.d.'s (except the e.s.d. in the dihedral angle between two l.s. planes) are estimated using the full covariance matrix. The cell e.s.d.'s are taken into account individually in the estimation of e.s.d.'s in distances, angles and torsion angles; correlations between e.s.d.'s in cell parameters are only used when they are defined by crystal symmetry. An approximate (isotropic) treatment of cell e.s.d.'s is used for estimating e.s.d.'s involving l.s. planes.

### Fractional atomic coordinates and isotropic or equivalent isotropic displacement parameters (Å^2^)

**Table:**

	*x*	*y*	*z*	*U*_iso_*/*U*_eq_	Occ. (<1)
Mo1	0.07030 (4)	0.98765 (2)	0.70745 (2)	0.01685 (8)	
Mo2	0.08559 (5)	0.86149 (3)	0.85537 (2)	0.02715 (10)	
Mo3	0.16217 (4)	0.60317 (3)	0.86796 (2)	0.02082 (9)	
Mo4	0.22287 (4)	0.47443 (2)	0.73545 (2)	0.01670 (8)	
Mo5	0.23106 (4)	0.60189 (2)	0.58822 (2)	0.01558 (8)	
Mo6	0.15245 (3)	0.85979 (2)	0.57545 (2)	0.01468 (8)	
O1	0.1659 (3)	0.9593 (2)	0.78943 (12)	0.0246 (6)	
O2	0.0207 (3)	0.7260 (2)	0.87992 (13)	0.0268 (7)	
O3	0.2976 (3)	0.5184 (2)	0.81276 (12)	0.0185 (5)	
O4	0.1380 (3)	0.5093 (2)	0.65144 (12)	0.0192 (6)	
O5	0.2965 (3)	0.7388 (2)	0.56585 (12)	0.0180 (5)	
O6	0.0066 (3)	0.9393 (2)	0.62919 (12)	0.0169 (5)	
O7	0.1890 (3)	1.0768 (2)	0.67652 (13)	0.0245 (6)	
O8	−0.0969 (3)	1.0558 (2)	0.73014 (13)	0.0274 (7)	
O9	0.2118 (5)	0.8721 (3)	0.91123 (14)	0.0443 (9)	
O1W	0.2590 (12)	0.9809 (15)	1.0154 (8)	0.184 (9)	0.5
H1WA	0.2462	0.9492	0.9832	0.276*	0.5
H1WB	0.2094	1.0047	1.0482	0.276*	0.5
O10	−0.0799 (4)	0.9283 (3)	0.88176 (15)	0.0438 (9)	
O11	0.2876 (4)	0.6120 (2)	0.92487 (14)	0.0346 (8)	
O12	0.0389 (4)	0.5191 (2)	0.90187 (14)	0.0314 (7)	
O13	0.0944 (3)	0.3921 (2)	0.76519 (14)	0.0299 (7)	
O14	0.3796 (3)	0.3960 (2)	0.71415 (14)	0.0274 (6)	
O15	0.1131 (3)	0.5942 (2)	0.52742 (13)	0.0230 (6)	
O16	0.3973 (3)	0.5344 (2)	0.56528 (13)	0.0226 (6)	
O17	0.2801 (3)	0.9456 (2)	0.54611 (12)	0.0206 (6)	
O18	0.0397 (3)	0.8525 (2)	0.51406 (12)	0.0215 (6)	
Fe1	0.16272 (5)	0.73185 (4)	0.72223 (2)	0.01210 (11)	
O1H	0.2536 (3)	0.7399 (2)	0.80532 (12)	0.0185 (6)	
H1H	0.3645	0.7472	0.8042	0.022*	
O2H	0.2464 (3)	0.85564 (19)	0.67550 (11)	0.0147 (5)	
H2H	0.3564	0.8633	0.6777	0.018*	
O3H	0.3128 (3)	0.62367 (19)	0.68738 (12)	0.0147 (5)	
H3H	0.4227	0.6314	0.6898	0.018*	
O4H	0.0625 (3)	0.7268 (2)	0.63805 (11)	0.0151 (5)	
H4H	−0.0486	0.7198	0.6402	0.018*	
O5H	0.0654 (3)	0.6135 (2)	0.76894 (12)	0.0161 (5)	
H5H	−0.0464	0.6125	0.7673	0.019*	
O6H	0.0001 (3)	0.8368 (2)	0.75643 (12)	0.0166 (5)	
H6H	−0.1079	0.8232	0.7553	0.020*	
S1	−0.33015 (11)	0.68657 (8)	0.74350 (5)	0.0269 (2)	
O1S	−0.2351 (3)	0.6113 (2)	0.78520 (15)	0.0332 (7)	
O2S	−0.2325 (3)	0.7606 (2)	0.70866 (15)	0.0349 (8)	
O3S	−0.3991 (3)	0.6318 (2)	0.69703 (15)	0.0312 (7)	
O4S	−0.4464 (3)	0.7430 (3)	0.78508 (17)	0.0405 (8)	
O1A	0.7444 (3)	0.6640 (2)	0.44577 (13)	0.0257 (6)	
C2A	0.7298 (5)	0.6408 (4)	0.5141 (2)	0.0291 (10)	
H2AA	0.8212	0.5969	0.5278	0.035*	
H2AB	0.6410	0.6018	0.5255	0.035*	
C3A	0.7109 (5)	0.7361 (4)	0.5492 (2)	0.0342 (11)	
H3AA	0.8029	0.7726	0.5407	0.041*	
H3AB	0.6962	0.7182	0.5964	0.041*	
N4A	0.5764 (4)	0.8037 (3)	0.52633 (17)	0.0291 (8)	
H4AA	0.5711	0.8649	0.5442	0.035*	
H4AB	0.4907	0.7738	0.5401	0.035*	
C5A	0.5837 (5)	0.8224 (3)	0.4542 (2)	0.0289 (9)	
H5AA	0.6676	0.8642	0.4391	0.035*	
H5AB	0.4877	0.8601	0.4404	0.035*	
C6A	0.6097 (5)	0.7196 (4)	0.4258 (2)	0.0295 (10)	
H6AA	0.5231	0.6796	0.4397	0.035*	
H6AB	0.6156	0.7302	0.3779	0.035*	
O1B	−0.3116 (3)	0.2911 (2)	0.65919 (15)	0.0287 (7)	
C2B	−0.2048 (4)	0.2376 (3)	0.6169 (2)	0.0257 (9)	
H2BA	−0.2334	0.2535	0.5718	0.031*	
H2BB	−0.2059	0.1627	0.6276	0.031*	
C3B	−0.0465 (4)	0.2670 (3)	0.6222 (2)	0.0222 (8)	
H3BA	−0.0151	0.2484	0.6667	0.027*	
H3BB	0.0263	0.2296	0.5918	0.027*	
N4B	−0.0472 (4)	0.3801 (2)	0.60629 (15)	0.0206 (7)	
H4BA	0.0454	0.3994	0.6124	0.025*	
H4BB	−0.0653	0.3957	0.5639	0.025*	
C5B	−0.1655 (4)	0.4378 (3)	0.6478 (2)	0.0246 (9)	
H5BA	−0.1708	0.5121	0.6340	0.030*	
H5BB	−0.1395	0.4280	0.6935	0.030*	
C6B	−0.3155 (5)	0.3992 (3)	0.6417 (2)	0.0311 (10)	
H6BA	−0.3938	0.4361	0.6701	0.037*	
H6BB	−0.3439	0.4138	0.5965	0.037*	
O1C	0.0475 (5)	0.1916 (5)	0.8976 (2)	0.0507 (14)	0.857 (6)
C2C	−0.0299 (8)	0.1850 (5)	0.8422 (3)	0.0418 (16)	0.857 (6)
H2CA	−0.0784	0.1203	0.8465	0.050*	0.857 (6)
H2CB	0.0437	0.1832	0.8038	0.050*	0.857 (6)
C3C	−0.1492 (6)	0.2742 (7)	0.8318 (3)	0.0405 (16)	0.857 (6)
H3CA	−0.1004	0.3386	0.8222	0.049*	0.857 (6)
H3CB	−0.2065	0.2643	0.7943	0.049*	0.857 (6)
N4C	−0.2543 (6)	0.2821 (5)	0.8907 (3)	0.030 (3)	0.857 (6)
H4CA	−0.3116	0.2284	0.8951	0.036*	0.857 (6)
H4CB	−0.3178	0.3411	0.8864	0.036*	0.857 (6)
C5C	−0.1696 (7)	0.2820 (6)	0.9505 (3)	0.0441 (17)	0.857 (6)
H5CA	−0.1188	0.3455	0.9493	0.053*	0.857 (6)
H5CB	−0.2406	0.2788	0.9896	0.053*	0.857 (6)
C6C	−0.0539 (8)	0.1896 (7)	0.9524 (3)	0.058 (2)	0.857 (6)
H6CA	0.0028	0.1877	0.9917	0.070*	0.857 (6)
H6CB	−0.1061	0.1264	0.9551	0.070*	0.857 (6)
O11C	−0.043 (3)	0.271 (2)	0.9549 (11)	0.057 (8)*	0.143 (6)
C21C	0.063 (3)	0.234 (3)	0.9067 (18)	0.061 (19)*	0.143 (6)
H21A	0.1521	0.1960	0.9271	0.074*	0.143 (6)
H21B	0.0988	0.2921	0.8782	0.074*	0.143 (6)
C31C	−0.007 (5)	0.163 (3)	0.867 (2)	0.10 (3)*	0.143 (6)
H31A	0.0682	0.1359	0.8336	0.122*	0.143 (6)
H31B	−0.0428	0.1046	0.8953	0.122*	0.143 (6)
N41C	−0.137 (4)	0.223 (3)	0.8359 (14)	0.053 (12)*	0.143 (6)
H41A	−0.1878	0.1801	0.8158	0.064*	0.143 (6)
H41B	−0.1014	0.2701	0.8053	0.064*	0.143 (6)
C51C	−0.245 (3)	0.278 (4)	0.884 (2)	0.07 (4)*	0.143 (6)
H51A	−0.3003	0.2272	0.9119	0.087*	0.143 (6)
H51B	−0.3194	0.3259	0.8609	0.087*	0.143 (6)
C61C	−0.158 (3)	0.3357 (19)	0.9249 (12)	0.020 (6)*	0.143 (6)
H61A	−0.1118	0.3913	0.8975	0.024*	0.143 (6)
H61B	−0.2282	0.3678	0.9585	0.024*	0.143 (6)
O1E	0.5358 (5)	1.1453 (3)	0.8694 (2)	0.0366 (10)	0.857 (6)
C2E	0.4163 (7)	1.1963 (5)	0.8317 (3)	0.0443 (16)	0.857 (6)
H2EA	0.4200	1.2716	0.8297	0.053*	0.857 (6)
H2EB	0.3168	1.1807	0.8528	0.053*	0.857 (6)
C3E	0.4328 (8)	1.1618 (4)	0.7643 (3)	0.0389 (15)	0.857 (6)
H3EA	0.5270	1.1840	0.7414	0.047*	0.857 (6)
H3EB	0.3461	1.1935	0.7399	0.047*	0.857 (6)
N4E	0.4379 (5)	1.0468 (4)	0.7675 (2)	0.0304 (11)	0.857 (6)
H4EA	0.3454	1.0277	0.7825	0.037*	0.857 (6)
H4EB	0.4586	1.0256	0.7268	0.037*	0.857 (6)
C5E	0.5540 (8)	0.9958 (5)	0.8100 (3)	0.0457 (17)	0.857 (6)
H5EA	0.6565	1.0085	0.7909	0.055*	0.857 (6)
H5EB	0.5476	0.9208	0.8146	0.055*	0.857 (6)
C6E	0.5269 (9)	1.0381 (5)	0.8748 (3)	0.0510 (19)	0.857 (6)
H6EA	0.4254	1.0235	0.8941	0.061*	0.857 (6)
H6EB	0.6033	1.0040	0.9039	0.061*	0.857 (6)
O11E	0.610 (3)	1.148 (2)	0.8423 (15)	0.048 (7)*	0.143 (6)
C21E	0.528 (4)	1.200 (3)	0.7879 (18)	0.044 (9)*	0.143 (6)
H21C	0.5806	1.1787	0.7466	0.053*	0.143 (6)
H21D	0.5273	1.2749	0.7883	0.053*	0.143 (6)
C31E	0.363 (4)	1.170 (3)	0.7931 (18)	0.029 (7)*	0.143 (6)
H31C	0.3099	1.1906	0.8344	0.035*	0.143 (6)
H31D	0.3071	1.2063	0.7570	0.035*	0.143 (6)
N41E	0.371 (3)	1.058 (2)	0.7901 (13)	0.026 (6)*	0.143 (6)
H41C	0.4108	1.0406	0.7504	0.032*	0.143 (6)
H41D	0.2757	1.0376	0.7961	0.032*	0.143 (6)
C51E	0.470 (3)	1.002 (2)	0.8430 (13)	0.016 (6)*	0.143 (6)
H51C	0.4192	1.0122	0.8864	0.019*	0.143 (6)
H51D	0.4835	0.9270	0.8376	0.019*	0.143 (6)
C61E	0.617 (4)	1.041 (3)	0.8376 (18)	0.038 (8)*	0.143 (6)
H61C	0.6784	1.0058	0.8721	0.046*	0.143 (6)
H61D	0.6700	1.0251	0.7954	0.046*	0.143 (6)
O1D	−0.4396 (8)	0.7161 (5)	1.0345 (3)	0.0377 (15)	0.703 (9)
C2D	−0.3989 (9)	0.7971 (5)	0.9881 (3)	0.039 (2)	0.703 (9)
H2DA	−0.3004	0.8185	0.9977	0.047*	0.703 (9)
H2DB	−0.4761	0.8570	0.9914	0.047*	0.703 (9)
C3D	−0.3873 (8)	0.7643 (6)	0.9214 (3)	0.0361 (19)	0.703 (9)
H3DA	−0.3537	0.8205	0.8906	0.043*	0.703 (9)
H3DB	−0.4882	0.7495	0.9101	0.043*	0.703 (9)
N4D	−0.2762 (9)	0.6697 (6)	0.9156 (3)	0.0303 (15)	0.703 (9)
H4DA	−0.2822	0.6448	0.8765	0.036*	0.703 (9)
H4DB	−0.1805	0.6869	0.9175	0.036*	0.703 (9)
C5D	−0.3069 (11)	0.5889 (6)	0.9678 (4)	0.041 (2)	0.703 (9)
H5DA	−0.2219	0.5336	0.9674	0.049*	0.703 (9)
H5DB	−0.4002	0.5587	0.9600	0.049*	0.703 (9)
C6D	−0.3255 (10)	0.6321 (6)	1.0326 (3)	0.046 (2)	0.703 (9)
H6DA	−0.3522	0.5776	1.0663	0.056*	0.703 (9)
H6DB	−0.2281	0.6549	1.0425	0.056*	0.703 (9)
O11D	−0.4846 (16)	0.7037 (12)	1.0209 (7)	0.027 (4)*	0.297 (9)
C21D	−0.4828 (19)	0.7728 (13)	0.9674 (8)	0.032 (4)*	0.297 (9)
H21E	−0.5345	0.8400	0.9789	0.038*	0.297 (9)
H21F	−0.5382	0.7483	0.9333	0.038*	0.297 (9)
C31D	−0.3180 (18)	0.7855 (11)	0.9415 (7)	0.025 (3)*	0.297 (9)
H31E	−0.3177	0.8357	0.9030	0.030*	0.297 (9)
H31F	−0.2638	0.8122	0.9751	0.030*	0.297 (9)
N41D	−0.241 (2)	0.6885 (15)	0.9241 (9)	0.027 (5)*	0.297 (9)
H41E	−0.1424	0.6955	0.9115	0.032*	0.297 (9)
H41F	−0.2853	0.6664	0.8906	0.032*	0.297 (9)
C51D	−0.252 (2)	0.6104 (15)	0.9834 (9)	0.033 (4)*	0.297 (9)
H51E	−0.2039	0.5422	0.9720	0.040*	0.297 (9)
H51F	−0.1978	0.6322	1.0188	0.040*	0.297 (9)
C61D	−0.4176 (16)	0.6035 (11)	1.0057 (7)	0.025 (4)*	0.297 (9)
H61E	−0.4711	0.5790	0.9710	0.030*	0.297 (9)
H61F	−0.4259	0.5543	1.0444	0.030*	0.297 (9)
O2W	0.5313 (3)	0.9178 (3)	0.67269 (17)	0.0394 (8)	
H2WA	0.5813	0.9500	0.6425	0.059*	
H2WB	0.5885	0.8684	0.6897	0.059*	
O3W	0.5901 (4)	0.4619 (3)	0.8460 (2)	0.0525 (11)	
H3WA	0.6398	0.4979	0.8176	0.079*	
H3WB	0.4967	0.4702	0.8386	0.079*	
O4W	−0.2744 (3)	0.9785 (3)	0.57958 (14)	0.0327 (7)	
H4WA	−0.2595	1.0042	0.5408	0.049*	
H4WB	−0.1942	0.9791	0.5993	0.049*	

### Atomic displacement parameters (Å^2^)

**Table:**

	*U*^11^	*U*^22^	*U*^33^	*U*^12^	*U*^13^	*U*^23^
Mo1	0.02266 (17)	0.01483 (17)	0.01282 (15)	0.00194 (12)	−0.00431 (12)	−0.00177 (12)
Mo2	0.0502 (2)	0.01917 (19)	0.01084 (16)	0.00373 (16)	−0.00339 (15)	−0.00225 (14)
Mo3	0.03058 (19)	0.01924 (18)	0.01175 (15)	0.00277 (14)	−0.00552 (13)	0.00067 (13)
Mo4	0.02022 (17)	0.01442 (17)	0.01648 (16)	−0.00286 (12)	−0.00614 (12)	−0.00127 (13)
Mo5	0.01895 (16)	0.01605 (17)	0.01302 (15)	−0.00288 (12)	−0.00493 (12)	−0.00354 (12)
Mo6	0.01686 (16)	0.01677 (17)	0.01099 (14)	−0.00214 (12)	−0.00403 (11)	−0.00110 (12)
O1	0.0416 (17)	0.0191 (15)	0.0147 (13)	−0.0020 (12)	−0.0111 (11)	−0.0027 (11)
O2	0.0430 (18)	0.0202 (15)	0.0137 (13)	0.0069 (13)	0.0033 (12)	0.0001 (11)
O3	0.0209 (13)	0.0187 (14)	0.0166 (12)	0.0005 (10)	−0.0082 (10)	−0.0019 (11)
O4	0.0245 (14)	0.0193 (14)	0.0157 (12)	−0.0051 (11)	−0.0072 (10)	−0.0030 (11)
O5	0.0194 (13)	0.0176 (14)	0.0170 (12)	−0.0020 (10)	0.0006 (10)	−0.0037 (11)
O6	0.0175 (13)	0.0188 (14)	0.0140 (12)	0.0016 (10)	−0.0040 (10)	−0.0014 (10)
O7	0.0348 (16)	0.0180 (15)	0.0213 (14)	−0.0024 (12)	−0.0085 (12)	−0.0005 (12)
O8	0.0317 (16)	0.0245 (16)	0.0231 (14)	0.0089 (12)	0.0011 (12)	−0.0028 (12)
O9	0.088 (3)	0.0299 (19)	0.0172 (15)	−0.0044 (18)	−0.0195 (16)	−0.0034 (13)
O1W	0.047 (6)	0.34 (2)	0.204 (15)	−0.034 (10)	0.009 (8)	−0.222 (16)
O10	0.073 (3)	0.0252 (18)	0.0257 (16)	0.0091 (16)	0.0178 (16)	0.0002 (14)
O11	0.056 (2)	0.0290 (17)	0.0201 (14)	0.0045 (15)	−0.0213 (14)	−0.0012 (13)
O12	0.0392 (18)	0.0264 (17)	0.0249 (15)	0.0032 (13)	0.0026 (13)	0.0052 (13)
O13	0.0352 (17)	0.0300 (17)	0.0277 (15)	−0.0146 (13)	−0.0124 (13)	0.0038 (13)
O14	0.0336 (16)	0.0221 (16)	0.0258 (15)	0.0034 (12)	−0.0055 (12)	−0.0035 (12)
O15	0.0272 (15)	0.0242 (15)	0.0198 (13)	−0.0051 (12)	−0.0099 (11)	−0.0040 (12)
O16	0.0241 (14)	0.0223 (15)	0.0219 (14)	−0.0012 (11)	−0.0027 (11)	−0.0060 (12)
O17	0.0211 (14)	0.0205 (15)	0.0195 (13)	−0.0003 (11)	−0.0007 (10)	−0.0001 (11)
O18	0.0253 (15)	0.0234 (15)	0.0163 (13)	−0.0007 (11)	−0.0062 (11)	−0.0028 (11)
Fe1	0.0122 (2)	0.0141 (3)	0.0106 (2)	−0.00148 (19)	−0.00381 (18)	−0.0014 (2)
O1H	0.0199 (13)	0.0204 (14)	0.0167 (12)	−0.0009 (11)	−0.0091 (10)	−0.0042 (11)
O2H	0.0148 (12)	0.0162 (13)	0.0139 (12)	−0.0024 (10)	−0.0046 (9)	−0.0015 (10)
O3H	0.0130 (12)	0.0162 (13)	0.0154 (12)	0.0002 (9)	−0.0038 (9)	−0.0041 (10)
O4H	0.0139 (12)	0.0194 (14)	0.0129 (12)	−0.0034 (10)	−0.0058 (9)	−0.0005 (10)
O5H	0.0134 (12)	0.0171 (14)	0.0183 (12)	−0.0038 (10)	−0.0063 (10)	0.0027 (11)
O6H	0.0148 (12)	0.0197 (14)	0.0145 (12)	0.0024 (10)	−0.0025 (9)	−0.0003 (11)
S1	0.0144 (5)	0.0299 (6)	0.0369 (6)	−0.0038 (4)	−0.0080 (4)	0.0019 (5)
O1S	0.0202 (15)	0.0391 (19)	0.0395 (18)	−0.0083 (13)	−0.0063 (13)	0.0112 (15)
O2S	0.0244 (15)	0.0367 (19)	0.0445 (19)	−0.0103 (13)	−0.0176 (13)	0.0150 (15)
O3S	0.0184 (14)	0.0361 (18)	0.0401 (18)	−0.0065 (12)	−0.0052 (12)	−0.0003 (14)
O4S	0.0203 (16)	0.046 (2)	0.060 (2)	−0.0082 (14)	−0.0074 (15)	−0.0161 (17)
O1A	0.0239 (15)	0.0290 (16)	0.0239 (14)	0.0007 (12)	−0.0022 (11)	−0.0054 (12)
C2A	0.026 (2)	0.035 (3)	0.026 (2)	−0.0006 (18)	−0.0054 (17)	0.0016 (19)
C3A	0.029 (2)	0.052 (3)	0.025 (2)	−0.011 (2)	−0.0061 (17)	−0.007 (2)
N4A	0.0265 (19)	0.031 (2)	0.0321 (19)	−0.0077 (15)	0.0075 (15)	−0.0162 (16)
C5A	0.026 (2)	0.025 (2)	0.034 (2)	0.0018 (17)	0.0024 (17)	0.0009 (19)
C6A	0.030 (2)	0.038 (3)	0.022 (2)	0.0001 (19)	−0.0096 (17)	−0.0072 (19)
O1B	0.0245 (15)	0.0247 (16)	0.0373 (17)	−0.0046 (12)	0.0005 (12)	−0.0047 (13)
C2B	0.023 (2)	0.023 (2)	0.031 (2)	−0.0014 (16)	−0.0095 (17)	−0.0026 (18)
C3B	0.023 (2)	0.019 (2)	0.025 (2)	−0.0010 (15)	−0.0053 (15)	−0.0004 (16)
N4B	0.0208 (16)	0.0244 (18)	0.0179 (15)	−0.0031 (13)	−0.0077 (12)	−0.0024 (14)
C5B	0.026 (2)	0.022 (2)	0.026 (2)	−0.0014 (16)	−0.0028 (16)	−0.0049 (17)
C6B	0.025 (2)	0.022 (2)	0.047 (3)	−0.0005 (17)	−0.0061 (19)	−0.007 (2)
O1C	0.036 (2)	0.072 (4)	0.040 (3)	0.013 (2)	−0.008 (2)	−0.002 (3)
C2C	0.054 (4)	0.035 (3)	0.031 (3)	0.011 (3)	0.011 (3)	−0.005 (3)
C3C	0.032 (3)	0.056 (5)	0.026 (3)	0.004 (3)	0.006 (2)	0.015 (3)
N4C	0.025 (3)	0.036 (4)	0.029 (3)	−0.0039 (18)	0.0011 (18)	−0.005 (2)
C5C	0.037 (3)	0.064 (5)	0.036 (3)	−0.009 (3)	0.001 (2)	−0.034 (3)
C6C	0.055 (4)	0.089 (6)	0.029 (3)	0.010 (4)	−0.016 (3)	−0.007 (3)
O1E	0.048 (3)	0.030 (2)	0.034 (2)	−0.0058 (18)	−0.019 (2)	−0.0044 (17)
C2E	0.049 (4)	0.033 (3)	0.054 (4)	−0.001 (3)	−0.021 (3)	−0.009 (3)
C3E	0.050 (4)	0.035 (3)	0.036 (3)	−0.021 (3)	−0.025 (3)	0.011 (3)
N4E	0.025 (2)	0.039 (3)	0.030 (2)	−0.009 (2)	−0.007 (2)	−0.006 (2)
C5E	0.050 (4)	0.033 (3)	0.058 (4)	0.002 (3)	−0.031 (3)	−0.008 (3)
C6E	0.085 (5)	0.034 (4)	0.038 (3)	−0.018 (3)	−0.030 (3)	0.010 (3)
O1D	0.041 (4)	0.044 (3)	0.022 (3)	0.012 (3)	0.002 (3)	0.006 (2)
C2D	0.048 (5)	0.030 (4)	0.036 (4)	0.007 (3)	0.001 (3)	−0.002 (3)
C3D	0.034 (4)	0.043 (4)	0.026 (3)	0.009 (3)	−0.002 (3)	0.010 (3)
N4D	0.035 (4)	0.037 (4)	0.019 (3)	−0.008 (3)	0.001 (3)	−0.005 (3)
C5D	0.063 (6)	0.024 (4)	0.033 (4)	0.003 (4)	0.002 (4)	0.001 (3)
C6D	0.059 (5)	0.044 (5)	0.029 (4)	0.017 (4)	−0.001 (3)	0.008 (3)
O2W	0.0255 (16)	0.044 (2)	0.049 (2)	−0.0111 (14)	−0.0154 (14)	0.0164 (17)
O3W	0.0206 (16)	0.064 (3)	0.068 (3)	−0.0085 (17)	−0.0126 (16)	0.032 (2)
O4W	0.0242 (15)	0.048 (2)	0.0244 (15)	−0.0006 (14)	−0.0078 (12)	0.0055 (15)

### Geometric parameters (Å, º)

**Table:**

Mo1—O1	1.954 (3)	N4C—H4CA	0.9100
Mo1—O6	1.944 (3)	N4C—H4CB	0.9100
Mo1—O7	1.712 (3)	N4C—C5C	1.503 (8)
Mo1—O8	1.705 (3)	C5C—H5CA	0.9900
Mo1—O2H	2.312 (3)	C5C—H5CB	0.9900
Mo1—O6H	2.273 (3)	C5C—C6C	1.503 (10)
Mo2—O1	1.942 (3)	C6C—H6CA	0.9900
Mo2—O2	1.944 (3)	C6C—H6CB	0.9900
Mo2—O9	1.703 (3)	O11C—C21C	1.404 (14)
Mo2—O10	1.706 (3)	O11C—C61C	1.403 (12)
Mo2—O1H	2.322 (3)	C21C—H21A	0.9900
Mo2—O6H	2.309 (3)	C21C—H21B	0.9900
Mo3—O2	1.957 (3)	C21C—C31C	1.511 (13)
Mo3—O3	1.928 (3)	C31C—H31A	0.9900
Mo3—O11	1.710 (3)	C31C—H31B	0.9900
Mo3—O12	1.710 (3)	C31C—N41C	1.484 (13)
Mo3—O1H	2.307 (3)	N41C—H41A	0.9100
Mo3—O5H	2.281 (3)	N41C—H41B	0.9100
Mo4—O3	1.944 (3)	N41C—C51C	1.500 (13)
Mo4—O4	1.955 (3)	C51C—H51A	0.9900
Mo4—O13	1.703 (3)	C51C—H51B	0.9900
Mo4—O14	1.694 (3)	C51C—C61C	1.501 (14)
Mo4—O3H	2.314 (3)	C61C—H61A	0.9900
Mo4—O5H	2.302 (3)	C61C—H61B	0.9900
Mo5—O4	1.912 (3)	O1E—C2E	1.442 (7)
Mo5—O5	1.957 (3)	O1E—C6E	1.413 (7)
Mo5—O15	1.723 (3)	C2E—H2EA	0.9900
Mo5—O16	1.696 (3)	C2E—H2EB	0.9900
Mo5—O3H	2.291 (3)	C2E—C3E	1.500 (9)
Mo5—O4H	2.351 (3)	C3E—H3EA	0.9900
Mo6—O5	1.944 (3)	C3E—H3EB	0.9900
Mo6—O6	1.923 (3)	C3E—N4E	1.504 (7)
Mo6—O17	1.727 (3)	N4E—H4EA	0.9100
Mo6—O18	1.701 (3)	N4E—H4EB	0.9100
Mo6—O2H	2.297 (2)	N4E—C5E	1.480 (7)
Mo6—O4H	2.264 (3)	C5E—H5EA	0.9900
O1W—H1WA	0.8397	C5E—H5EB	0.9900
O1W—H1WB	0.8468	C5E—C6E	1.493 (10)
Fe1—O1H	1.981 (3)	C6E—H6EA	0.9900
Fe1—O2H	2.003 (3)	C6E—H6EB	0.9900
Fe1—O3H	1.985 (3)	O11E—C21E	1.47 (4)
Fe1—O4H	2.036 (3)	O11E—C61E	1.41 (5)
Fe1—O5H	2.001 (3)	C21E—H21C	0.9900
Fe1—O6H	2.018 (3)	C21E—H21D	0.9900
O1H—H1H	1.0000	C21E—C31E	1.55 (5)
O2H—H2H	1.0000	C31E—H31C	0.9900
O3H—H3H	1.0000	C31E—H31D	0.9900
O4H—H4H	1.0000	C31E—N41E	1.48 (4)
O5H—H5H	1.0000	N41E—H41C	0.9100
O6H—H6H	1.0000	N41E—H41D	0.9100
S1—O1S	1.493 (3)	N41E—C51E	1.55 (4)
S1—O2S	1.478 (3)	C51E—H51C	0.9900
S1—O3S	1.464 (3)	C51E—H51D	0.9900
S1—O4S	1.473 (4)	C51E—C61E	1.45 (4)
O1A—C2A	1.423 (5)	C61E—H61C	0.9900
O1A—C6A	1.406 (5)	C61E—H61D	0.9900
C2A—H2AA	0.9900	O1D—C2D	1.430 (9)
C2A—H2AB	0.9900	O1D—C6D	1.417 (9)
C2A—C3A	1.492 (7)	C2D—H2DA	0.9900
C3A—H3AA	0.9900	C2D—H2DB	0.9900
C3A—H3AB	0.9900	C2D—C3D	1.479 (10)
C3A—N4A	1.494 (6)	C3D—H3DA	0.9900
N4A—H4AA	0.9100	C3D—H3DB	0.9900
N4A—H4AB	0.9100	C3D—N4D	1.508 (10)
N4A—C5A	1.494 (5)	N4D—H4DA	0.9100
C5A—H5AA	0.9900	N4D—H4DB	0.9100
C5A—H5AB	0.9900	N4D—C5D	1.480 (10)
C5A—C6A	1.510 (6)	C5D—H5DA	0.9900
C6A—H6AA	0.9900	C5D—H5DB	0.9900
C6A—H6AB	0.9900	C5D—C6D	1.496 (11)
O1B—C2B	1.413 (5)	C6D—H6DA	0.9900
O1B—C6B	1.432 (5)	C6D—H6DB	0.9900
C2B—H2BA	0.9900	O11D—C21D	1.37 (2)
C2B—H2BB	0.9900	O11D—C61D	1.44 (2)
C2B—C3B	1.516 (5)	C21D—H21E	0.9900
C3B—H3BA	0.9900	C21D—H21F	0.9900
C3B—H3BB	0.9900	C21D—C31D	1.54 (2)
C3B—N4B	1.494 (5)	C31D—H31E	0.9900
N4B—H4BA	0.9100	C31D—H31F	0.9900
N4B—H4BB	0.9100	C31D—N41D	1.44 (2)
N4B—C5B	1.491 (5)	N41D—H41E	0.9100
C5B—H5BA	0.9900	N41D—H41F	0.9100
C5B—H5BB	0.9900	N41D—C51D	1.54 (3)
C5B—C6B	1.497 (6)	C51D—H51E	0.9900
C6B—H6BA	0.9900	C51D—H51F	0.9900
C6B—H6BB	0.9900	C51D—C61D	1.52 (2)
O1C—C2C	1.403 (9)	C61D—H61E	0.9900
O1C—C6C	1.395 (8)	C61D—H61F	0.9900
C2C—H2CA	0.9900	O2W—H2WA	0.8486
C2C—H2CB	0.9900	O2W—H2WB	0.8488
C2C—C3C	1.505 (9)	O3W—H3WA	0.8504
C3C—H3CA	0.9900	O3W—H3WB	0.8517
C3C—H3CB	0.9900	O4W—H4WA	0.8512
C3C—N4C	1.484 (8)	O4W—H4WB	0.8530
			
O1—Mo1—O2H	82.96 (11)	C5B—N4B—H4BB	109.5
O1—Mo1—O6H	71.17 (11)	N4B—C5B—H5BA	109.9
O6—Mo1—O1	149.82 (11)	N4B—C5B—H5BB	109.9
O6—Mo1—O2H	71.81 (10)	N4B—C5B—C6B	109.1 (3)
O6—Mo1—O6H	84.95 (10)	H5BA—C5B—H5BB	108.3
O7—Mo1—O1	94.58 (13)	C6B—C5B—H5BA	109.9
O7—Mo1—O6	101.90 (12)	C6B—C5B—H5BB	109.9
O7—Mo1—O2H	90.82 (12)	O1B—C6B—C5B	111.8 (3)
O7—Mo1—O6H	158.02 (11)	O1B—C6B—H6BA	109.3
O8—Mo1—O1	102.23 (13)	O1B—C6B—H6BB	109.3
O8—Mo1—O6	97.36 (13)	C5B—C6B—H6BA	109.3
O8—Mo1—O7	105.90 (14)	C5B—C6B—H6BB	109.3
O8—Mo1—O2H	161.86 (13)	H6BA—C6B—H6BB	107.9
O8—Mo1—O6H	93.71 (12)	C6C—O1C—C2C	109.8 (5)
O6H—Mo1—O2H	71.30 (9)	O1C—C2C—H2CA	109.2
O1—Mo2—O2	149.50 (12)	O1C—C2C—H2CB	109.2
O1—Mo2—O1H	85.23 (11)	O1C—C2C—C3C	112.0 (5)
O1—Mo2—O6H	70.54 (11)	H2CA—C2C—H2CB	107.9
O2—Mo2—O1H	71.10 (11)	C3C—C2C—H2CA	109.2
O2—Mo2—O6H	83.48 (11)	C3C—C2C—H2CB	109.2
O9—Mo2—O1	96.60 (15)	C2C—C3C—H3CA	109.7
O9—Mo2—O2	102.52 (15)	C2C—C3C—H3CB	109.7
O9—Mo2—O10	105.99 (18)	H3CA—C3C—H3CB	108.2
O9—Mo2—O1H	90.80 (14)	N4C—C3C—C2C	109.7 (5)
O9—Mo2—O6H	158.22 (14)	N4C—C3C—H3CA	109.7
O10—Mo2—O1	101.56 (14)	N4C—C3C—H3CB	109.7
O10—Mo2—O2	95.77 (15)	C3C—N4C—H4CA	109.3
O10—Mo2—O1H	160.83 (15)	C3C—N4C—H4CB	109.3
O10—Mo2—O6H	94.04 (15)	C3C—N4C—C5C	111.5 (5)
O6H—Mo2—O1H	71.13 (9)	H4CA—N4C—H4CB	108.0
O2—Mo3—O1H	71.25 (11)	C5C—N4C—H4CA	109.3
O2—Mo3—O5H	83.28 (11)	C5C—N4C—H4CB	109.3
O3—Mo3—O2	150.44 (11)	N4C—C5C—H5CA	110.1
O3—Mo3—O1H	85.43 (11)	N4C—C5C—H5CB	110.1
O3—Mo3—O5H	71.97 (10)	H5CA—C5C—H5CB	108.4
O11—Mo3—O2	101.54 (14)	C6C—C5C—N4C	108.1 (5)
O11—Mo3—O3	96.56 (14)	C6C—C5C—H5CA	110.1
O11—Mo3—O1H	90.81 (13)	C6C—C5C—H5CB	110.1
O11—Mo3—O5H	159.23 (13)	O1C—C6C—C5C	111.3 (6)
O12—Mo3—O2	94.92 (14)	O1C—C6C—H6CA	109.4
O12—Mo3—O3	102.24 (13)	O1C—C6C—H6CB	109.4
O12—Mo3—O11	106.25 (15)	C5C—C6C—H6CA	109.4
O12—Mo3—O1H	160.13 (12)	C5C—C6C—H6CB	109.4
O12—Mo3—O5H	93.31 (13)	H6CA—C6C—H6CB	108.0
O5H—Mo3—O1H	71.43 (9)	C61C—O11C—C21C	108.7 (15)
O3—Mo4—O4	149.43 (11)	O11C—C21C—H21A	109.7
O3—Mo4—O3H	83.18 (10)	O11C—C21C—H21B	109.7
O3—Mo4—O5H	71.25 (10)	O11C—C21C—C31C	109.8 (16)
O4—Mo4—O3H	70.39 (10)	H21A—C21C—H21B	108.2
O4—Mo4—O5H	85.41 (11)	C31C—C21C—H21A	109.7
O13—Mo4—O3	103.68 (13)	C31C—C21C—H21B	109.7
O13—Mo4—O4	96.24 (13)	C21C—C31C—H31A	110.1
O13—Mo4—O3H	158.31 (12)	C21C—C31C—H31B	110.1
O13—Mo4—O5H	91.61 (13)	H31A—C31C—H31B	108.4
O14—Mo4—O3	96.60 (13)	N41C—C31C—C21C	108.1 (15)
O14—Mo4—O4	100.88 (13)	N41C—C31C—H31A	110.1
O14—Mo4—O13	103.89 (15)	N41C—C31C—H31B	110.1
O14—Mo4—O3H	95.57 (12)	C31C—N41C—H41A	109.2
O14—Mo4—O5H	162.40 (12)	C31C—N41C—H41B	109.2
O5H—Mo4—O3H	70.87 (9)	C31C—N41C—C51C	112.1 (16)
O4—Mo5—O5	147.96 (11)	H41A—N41C—H41B	107.9
O4—Mo5—O3H	71.61 (10)	C51C—N41C—H41A	109.2
O4—Mo5—O4H	83.53 (11)	C51C—N41C—H41B	109.2
O5—Mo5—O3H	83.23 (10)	N41C—C51C—H51A	109.7
O5—Mo5—O4H	69.51 (10)	N41C—C51C—H51B	109.8
O15—Mo5—O4	97.32 (12)	N41C—C51C—C61C	109.6 (15)
O15—Mo5—O5	100.87 (12)	H51A—C51C—H51B	108.2
O15—Mo5—O3H	161.18 (11)	C61C—C51C—H51A	109.7
O15—Mo5—O4H	92.81 (12)	C61C—C51C—H51B	109.8
O16—Mo5—O4	103.40 (13)	O11C—C61C—C51C	111.3 (16)
O16—Mo5—O5	96.86 (12)	O11C—C61C—H61A	109.4
O16—Mo5—O15	105.52 (14)	O11C—C61C—H61B	109.4
O16—Mo5—O3H	92.07 (12)	C51C—C61C—H61A	109.4
O16—Mo5—O4H	159.17 (12)	C51C—C61C—H61B	109.4
O3H—Mo5—O4H	71.24 (9)	H61A—C61C—H61B	108.0
O5—Mo6—O2H	83.57 (10)	C6E—O1E—C2E	109.8 (5)
O5—Mo6—O4H	71.69 (10)	O1E—C2E—H2EA	109.5
O6—Mo6—O5	149.28 (10)	O1E—C2E—H2EB	109.5
O6—Mo6—O2H	72.51 (10)	O1E—C2E—C3E	110.5 (5)
O6—Mo6—O4H	82.58 (11)	H2EA—C2E—H2EB	108.1
O17—Mo6—O5	94.63 (12)	C3E—C2E—H2EA	109.5
O17—Mo6—O6	103.69 (12)	C3E—C2E—H2EB	109.5
O17—Mo6—O2H	88.87 (11)	C2E—C3E—H3EA	109.8
O17—Mo6—O4H	157.37 (11)	C2E—C3E—H3EB	109.8
O18—Mo6—O5	101.37 (12)	C2E—C3E—N4E	109.5 (5)
O18—Mo6—O6	97.17 (12)	H3EA—C3E—H3EB	108.2
O18—Mo6—O17	105.74 (13)	N4E—C3E—H3EA	109.8
O18—Mo6—O2H	163.99 (11)	N4E—C3E—H3EB	109.8
O18—Mo6—O4H	94.82 (12)	C3E—N4E—H4EA	109.2
O4H—Mo6—O2H	72.08 (9)	C3E—N4E—H4EB	109.2
Mo2—O1—Mo1	119.53 (15)	H4EA—N4E—H4EB	107.9
Mo2—O2—Mo3	120.08 (15)	C5E—N4E—C3E	111.9 (4)
Mo3—O3—Mo4	118.91 (13)	C5E—N4E—H4EA	109.2
Mo5—O4—Mo4	120.31 (14)	C5E—N4E—H4EB	109.2
Mo6—O5—Mo5	119.87 (13)	N4E—C5E—H5EA	110.0
Mo6—O6—Mo1	118.99 (13)	N4E—C5E—H5EB	110.0
H1WA—O1W—H1WB	140.1	N4E—C5E—C6E	108.7 (5)
O1H—Fe1—O2H	96.79 (11)	H5EA—C5E—H5EB	108.3
O1H—Fe1—O3H	97.53 (11)	C6E—C5E—H5EA	110.0
O1H—Fe1—O4H	177.91 (10)	C6E—C5E—H5EB	110.0
O1H—Fe1—O5H	84.54 (11)	O1E—C6E—C5E	110.9 (5)
O1H—Fe1—O6H	84.69 (11)	O1E—C6E—H6EA	109.5
O2H—Fe1—O4H	83.25 (10)	O1E—C6E—H6EB	109.5
O2H—Fe1—O6H	83.30 (11)	C5E—C6E—H6EA	109.5
O3H—Fe1—O2H	99.02 (11)	C5E—C6E—H6EB	109.5
O3H—Fe1—O4H	84.52 (11)	H6EA—C6E—H6EB	108.0
O3H—Fe1—O5H	84.35 (11)	C61E—O11E—C21E	108 (3)
O3H—Fe1—O6H	176.55 (10)	O11E—C21E—H21C	109.6
O5H—Fe1—O2H	176.15 (10)	O11E—C21E—H21D	109.6
O5H—Fe1—O4H	95.29 (11)	O11E—C21E—C31E	110 (3)
O5H—Fe1—O6H	93.25 (11)	H21C—C21E—H21D	108.1
O6H—Fe1—O4H	93.25 (11)	C31E—C21E—H21C	109.6
Mo2—O1H—H1H	118.2	C31E—C21E—H21D	109.6
Mo3—O1H—Mo2	93.80 (10)	C21E—C31E—H31C	110.2
Mo3—O1H—H1H	118.2	C21E—C31E—H31D	110.2
Fe1—O1H—Mo2	102.45 (11)	H31C—C31E—H31D	108.5
Fe1—O1H—Mo3	101.86 (11)	N41E—C31E—C21E	107 (3)
Fe1—O1H—H1H	118.2	N41E—C31E—H31C	110.2
Mo1—O2H—H2H	118.5	N41E—C31E—H31D	110.2
Mo6—O2H—Mo1	92.61 (9)	C31E—N41E—H41C	109.6
Mo6—O2H—H2H	118.5	C31E—N41E—H41D	109.6
Fe1—O2H—Mo1	102.24 (10)	C31E—N41E—C51E	110 (2)
Fe1—O2H—Mo6	102.24 (11)	H41C—N41E—H41D	108.1
Fe1—O2H—H2H	118.5	C51E—N41E—H41C	109.6
Mo4—O3H—H3H	117.7	C51E—N41E—H41D	109.6
Mo5—O3H—Mo4	93.52 (10)	N41E—C51E—H51C	109.7
Mo5—O3H—H3H	117.7	N41E—C51E—H51D	109.7
Fe1—O3H—Mo4	102.43 (10)	H51C—C51E—H51D	108.2
Fe1—O3H—Mo5	103.95 (11)	C61E—C51E—N41E	110 (2)
Fe1—O3H—H3H	117.7	C61E—C51E—H51C	109.7
Mo5—O4H—H4H	118.6	C61E—C51E—H51D	109.7
Mo6—O4H—Mo5	94.02 (9)	O11E—C61E—C51E	114 (3)
Mo6—O4H—H4H	118.6	O11E—C61E—H61C	108.7
Fe1—O4H—Mo5	100.27 (10)	O11E—C61E—H61D	108.7
Fe1—O4H—Mo6	102.34 (11)	C51E—C61E—H61C	108.7
Fe1—O4H—H4H	118.6	C51E—C61E—H61D	108.7
Mo3—O5H—Mo4	93.37 (9)	H61C—C61E—H61D	107.6
Mo3—O5H—H5H	118.3	C6D—O1D—C2D	109.7 (6)
Mo4—O5H—H5H	118.3	O1D—C2D—H2DA	109.4
Fe1—O5H—Mo3	102.11 (11)	O1D—C2D—H2DB	109.4
Fe1—O5H—Mo4	102.35 (11)	O1D—C2D—C3D	111.3 (6)
Fe1—O5H—H5H	118.3	H2DA—C2D—H2DB	108.0
Mo1—O6H—Mo2	94.54 (10)	C3D—C2D—H2DA	109.4
Mo1—O6H—H6H	117.9	C3D—C2D—H2DB	109.4
Mo2—O6H—H6H	117.9	C2D—C3D—H3DA	109.5
Fe1—O6H—Mo1	103.15 (11)	C2D—C3D—H3DB	109.5
Fe1—O6H—Mo2	101.72 (11)	C2D—C3D—N4D	110.8 (6)
Fe1—O6H—H6H	117.9	H3DA—C3D—H3DB	108.1
O2S—S1—O1S	108.74 (16)	N4D—C3D—H3DA	109.5
O3S—S1—O1S	109.32 (19)	N4D—C3D—H3DB	109.5
O3S—S1—O2S	109.95 (19)	C3D—N4D—H4DA	109.3
O3S—S1—O4S	111.28 (18)	C3D—N4D—H4DB	109.3
O4S—S1—O1S	108.9 (2)	H4DA—N4D—H4DB	108.0
O4S—S1—O2S	108.6 (2)	C5D—N4D—C3D	111.6 (6)
C6A—O1A—C2A	109.2 (3)	C5D—N4D—H4DA	109.3
O1A—C2A—H2AA	109.4	C5D—N4D—H4DB	109.3
O1A—C2A—H2AB	109.4	N4D—C5D—H5DA	109.5
O1A—C2A—C3A	111.3 (4)	N4D—C5D—H5DB	109.5
H2AA—C2A—H2AB	108.0	N4D—C5D—C6D	110.8 (7)
C3A—C2A—H2AA	109.4	H5DA—C5D—H5DB	108.1
C3A—C2A—H2AB	109.4	C6D—C5D—H5DA	109.5
C2A—C3A—H3AA	109.9	C6D—C5D—H5DB	109.5
C2A—C3A—H3AB	109.9	O1D—C6D—C5D	111.9 (6)
C2A—C3A—N4A	109.1 (3)	O1D—C6D—H6DA	109.2
H3AA—C3A—H3AB	108.3	O1D—C6D—H6DB	109.2
N4A—C3A—H3AA	109.9	C5D—C6D—H6DA	109.2
N4A—C3A—H3AB	109.9	C5D—C6D—H6DB	109.2
C3A—N4A—H4AA	109.2	H6DA—C6D—H6DB	107.9
C3A—N4A—H4AB	109.2	C21D—O11D—C61D	111.7 (13)
C3A—N4A—C5A	112.1 (3)	O11D—C21D—H21E	109.6
H4AA—N4A—H4AB	107.9	O11D—C21D—H21F	109.6
C5A—N4A—H4AA	109.2	O11D—C21D—C31D	110.1 (13)
C5A—N4A—H4AB	109.2	H21E—C21D—H21F	108.2
N4A—C5A—H5AA	110.1	C31D—C21D—H21E	109.6
N4A—C5A—H5AB	110.1	C31D—C21D—H21F	109.6
N4A—C5A—C6A	108.1 (3)	C21D—C31D—H31E	109.6
H5AA—C5A—H5AB	108.4	C21D—C31D—H31F	109.6
C6A—C5A—H5AA	110.1	H31E—C31D—H31F	108.1
C6A—C5A—H5AB	110.1	N41D—C31D—C21D	110.4 (14)
O1A—C6A—C5A	111.0 (3)	N41D—C31D—H31E	109.6
O1A—C6A—H6AA	109.4	N41D—C31D—H31F	109.6
O1A—C6A—H6AB	109.4	C31D—N41D—H41E	110.0
C5A—C6A—H6AA	109.4	C31D—N41D—H41F	110.0
C5A—C6A—H6AB	109.4	C31D—N41D—C51D	108.4 (16)
H6AA—C6A—H6AB	108.0	H41E—N41D—H41F	108.4
C2B—O1B—C6B	109.5 (3)	C51D—N41D—H41E	110.0
O1B—C2B—H2BA	109.4	C51D—N41D—H41F	110.0
O1B—C2B—H2BB	109.4	N41D—C51D—H51E	109.8
O1B—C2B—C3B	111.1 (3)	N41D—C51D—H51F	109.8
H2BA—C2B—H2BB	108.0	H51E—C51D—H51F	108.3
C3B—C2B—H2BA	109.4	C61D—C51D—N41D	109.2 (15)
C3B—C2B—H2BB	109.4	C61D—C51D—H51E	109.8
C2B—C3B—H3BA	110.0	C61D—C51D—H51F	109.8
C2B—C3B—H3BB	110.0	O11D—C61D—C51D	108.5 (13)
H3BA—C3B—H3BB	108.3	O11D—C61D—H61E	110.0
N4B—C3B—C2B	108.7 (3)	O11D—C61D—H61F	110.0
N4B—C3B—H3BA	110.0	C51D—C61D—H61E	110.0
N4B—C3B—H3BB	110.0	C51D—C61D—H61F	110.0
C3B—N4B—H4BA	109.5	H61E—C61D—H61F	108.4
C3B—N4B—H4BB	109.5	H2WA—O2W—H2WB	109.7
H4BA—N4B—H4BB	108.0	H3WA—O3W—H3WB	109.3
C5B—N4B—C3B	110.9 (3)	H4WA—O4W—H4WB	109.1
C5B—N4B—H4BA	109.5		
			
O1A—C2A—C3A—N4A	56.9 (4)	O1E—C2E—C3E—N4E	55.3 (7)
C2A—O1A—C6A—C5A	64.4 (4)	C2E—O1E—C6E—C5E	64.1 (7)
C2A—C3A—N4A—C5A	−52.4 (5)	C2E—C3E—N4E—C5E	−52.5 (7)
C3A—N4A—C5A—C6A	52.8 (5)	C3E—N4E—C5E—C6E	53.9 (7)
N4A—C5A—C6A—O1A	−58.7 (4)	N4E—C5E—C6E—O1E	−59.7 (7)
C6A—O1A—C2A—C3A	−63.6 (4)	C6E—O1E—C2E—C3E	−61.8 (7)
O1B—C2B—C3B—N4B	−58.7 (4)	O11E—C21E—C31E—N41E	−61 (4)
C2B—O1B—C6B—C5B	−61.6 (5)	C21E—O11E—C61E—C51E	−63 (4)
C2B—C3B—N4B—C5B	54.7 (4)	C21E—C31E—N41E—C51E	55 (3)
C3B—N4B—C5B—C6B	−54.4 (4)	C31E—N41E—C51E—C61E	−53 (3)
N4B—C5B—C6B—O1B	57.5 (5)	N41E—C51E—C61E—O11E	57 (4)
C6B—O1B—C2B—C3B	61.9 (4)	C61E—O11E—C21E—C31E	63 (4)
O1C—C2C—C3C—N4C	−55.1 (7)	O1D—C2D—C3D—N4D	−56.2 (9)
C2C—O1C—C6C—C5C	−64.2 (8)	C2D—O1D—C6D—C5D	−61.6 (9)
C2C—C3C—N4C—C5C	51.1 (8)	C2D—C3D—N4D—C5D	49.8 (10)
C3C—N4C—C5C—C6C	−52.9 (8)	C3D—N4D—C5D—C6D	−48.9 (10)
N4C—C5C—C6C—O1C	59.4 (8)	N4D—C5D—C6D—O1D	55.4 (10)
C6C—O1C—C2C—C3C	61.6 (8)	C6D—O1D—C2D—C3D	62.2 (8)
O11C—C21C—C31C—N41C	61 (3)	O11D—C21D—C31D—N41D	59.6 (19)
C21C—O11C—C61C—C51C	65 (3)	C21D—O11D—C61D—C51D	63.0 (17)
C21C—C31C—N41C—C51C	−52 (3)	C21D—C31D—N41D—C51D	−55.8 (18)
C31C—N41C—C51C—C61C	50 (4)	C31D—N41D—C51D—C61D	57.0 (19)
N41C—C51C—C61C—O11C	−55 (3)	N41D—C51D—C61D—O11D	−58.6 (18)
C61C—O11C—C21C—C31C	−67 (3)	C61D—O11D—C21D—C31D	−62.2 (17)

### Hydrogen-bond geometry (Å, º)

**Table:**

*D*—H···*A*	*D*—H	H···*A*	*D*···*A*	*D*—H···*A*
O1*W*—H1*WA*···O9	0.84	1.94	2.781 (11)	178
O1*W*—H1*WB*···O10^i^	0.85	1.99	2.831 (11)	173
O2*W*—H2*WA*···O4*W*^ii^	0.85	1.80	2.619 (4)	161
O2*W*—H2*WB*···O2*S*^ii^	0.85	2.05	2.876 (4)	164
O2*W*—H2*WB*···O4*S*^ii^	0.85	2.48	3.129 (5)	133
O3*W*—H3*WA*···O1*S*^ii^	0.85	1.99	2.790 (5)	155
O3*W*—H3*WB*···O3	0.85	1.92	2.753 (4)	167
O4*W*—H4*WA*···O17^iii^	0.85	1.88	2.714 (4)	165
O4*W*—H4*WB*···O6	0.85	1.94	2.761 (4)	161
O1*H*—H1*H*···O4*S*^ii^	1.00	1.69	2.673 (4)	165
O2*H*—H2*H*···O2*W*	1.00	1.78	2.743 (4)	162
O3*H*—H3*H*···O3*S*^ii^	1.00	1.61	2.602 (4)	174
O4*H*—H4*H*···O2*S*	1.00	2.13	2.911 (4)	133
O5*H*—H5*H*···O1*S*	1.00	1.69	2.672 (4)	165
O6*H*—H6*H*···O2*S*	1.00	1.83	2.691 (4)	142
N4*A*—H4*AA*···O4*W*^ii^	0.91	2.34	3.102 (5)	141
N4*A*—H4*AB*···O5	0.91	1.86	2.760 (4)	169
N4*B*—H4*BA*···O4	0.91	2.00	2.761 (4)	140
N4*B*—H4*BA*···O1*A*^iv^	0.91	2.26	2.840 (4)	121
N4*B*—H4*BB*···O15^v^	0.91	1.97	2.869 (4)	171
N4*C*—H4*CA*···O1*E*^vi^	0.91	1.97	2.817 (7)	155
N41*C*—H41*A*···O11*E*^vi^	0.91	1.92	2.54 (5)	124
N4*E*—H4*EA*···O1	0.91	1.91	2.780 (6)	160
N41*E*—H41*D*···O1	0.91	1.52	2.35 (3)	150
N4*D*—H4*DA*···O1*S*	0.91	1.99	2.866 (8)	162
N4*D*—H4*DB*···O2	0.91	2.00	2.846 (9)	155
N41*D*—H41*E*···O2	0.91	1.62	2.520 (19)	169

Symmetry codes: (i) −*x*, −*y*+2, −*z*+2; (ii) *x*+1, *y*, *z*; (iii) −*x*, −*y*+2, −*z*+1; (iv) −*x*+1, −*y*+1, −*z*+1; (v) −*x*, −*y*+1, −*z*+1; (vi) *x*−1, *y*−1, *z*.
